# State of sustainability assessments of metal additive manufacturing: literature review and triple-bottom-line perspectives

**DOI:** 10.1007/s11356-025-36889-0

**Published:** 2025-08-28

**Authors:** Nur Safirah Bahuriddin, Muhamad Zameri Mat Saman, Nurhasyimah Mohamad-Ali, Nor Hasrul Akhmal Ngadiman, Muhd Ikmal Isyraf Mohd Maulana

**Affiliations:** 1 Faculty of Mechanical Engineering, Universiti Teknologi Malaysia, 81310 Bahru, Johor, Malaysia; 2 Research Management Centre, Level 1, F54, Building School Graduate Student, Universiti Teknologi Malaysia, 81310 Bahru, Johor, Malaysia

**Keywords:** Sustainability assessment, Additive manufacturing, Triple bottom line, Circular economy, Sustainable manufacturing, Recycling, Sustainability indicator

## Abstract

With growing global energy and climate concerns, metal additive manufacturing (AM) has emerged as an innovative solution, offering various advantages, particularly in minimizing environmental effects. However, the sustainability assessment of metal AM, in particular, environmental, economic, and social impacts under the triple-bottom-line (TBL) has not been adequately communicated to guarantee high-quality outcomes. This study reviews emerging sustainability assessment of metal AM, focusing on papers addressing multiple sustainability dimensions. Several parameters are critically analyzed, including sustainability dimensions, sustainability indicators, circular economy domain, life cycle stages, metal AM technology, and others. The findings indicate that life cycle assessment and life cycle costing are the most commonly used methods; however, both are applied independently, with no effort to integrate them into a single sustainability score. Besides, the environmental dimension is the primary consideration, while the economic and social dimension lacks sufficient exploration. From the perspective of life cycle stages, the AM stage is the primary focus within the system boundary, whereas the design, use and end-of-life stages are underrepresented. Resource efficiency is covered within the circular economy domain, while recycling, a fundamental aspect of metal AM with considerable potential, remains overlooked. This study serves as a valuable reference to enhance the inclusivity of sustainability assessments of metal AM, focusing on integrating TBL, life cycle stages, indicator reliability and recycling aspects.

## Introduction

The rapid expansion of manufacturing industries has substantially governed the economic system worldwide in reference to value-added for the growth of industrial metabolism. The proliferated intensity of development in these industries contributes to meeting the demand for products and services. However, this growth also has negative repercussions, such as depleting natural resources and causing harm to the environment (Peng et al. 2018). For the past two decades, the manufacturing industries have accounted for about 15% of global energy consumption and 35% of global material consumption (Hegab et al. 2023). The global sustainability issues could be notably improved with exertion from industrial manufacturing to reduce the waste, emissions and the use of energy and material (Jayawardane et al. 2023).

An increasing number of researchers prioritize sustainability as a crucial objective within manufacturing and engineering (Lee et al. 2019). As this manufacturing industry is driven by the demands for high quality of life and demographic rise, it consumes substantial resources and contributes significantly to pollution generation (Cassettari et al. 2017). Sun et al. (2020) mentioned that the manufacturing industry has become a major contributor to the resource utilization and emissions of carbon compounds. Given that manufacturing processes are pivotal in producing essential products that enhance the quality of human life and bolster the global economy, it is crucial to analyse these processes through the lens of sustainability.

According to Rosen and Kishawy (2012), sustainable manufacturing refers to the process of generating manufactured outputs via economically cost-effective approaches that reduce the impacts on the environment while simultaneously preserving energy and the earth’s resources. This terminology is tightly connected with triple-bottom-line (TBL) assessment as it considers the environmental, economic and social dimensions in the manufacturing concepts (Sartal et al. 2020). The TBL assessment enforces environmental and social roles by including supplementary performance metrics in the economic pillar (Gao and Zhang 2006).

The emergence of advanced manufacturing industries, mostly driven by additive manufacturing (AM) or 3D printing, is closely linked to the growing demand for resource efficiency in small-scale production (Ford and Despeisse 2016) and yielding immense enhancements in terms of excellence, cost-effectiveness and processing time (Agnusdei and Del Prete 2022). Besides, the capacity of AM to minimize material waste and fabricate lightweight components results in significant material efficiency gains during the manufacturing workflow, which employs the layering technique of 3D production (Elsacker et al. 2022). The near-net shape capability of AM significantly reduces the need for extensive post-processing and machining, thereby conserving materials (DebRoy et al. 2018). Furthermore, AM enables the fabrication of complex and lightweight structures, such as lattice geometries and topology-optimized components, which maintain mechanical strength while using less material (Thompson et al. 2016). From an environmental perspective, less material usage translates to lower resource extraction and reduced lifecycle emissions. Besides, material efficiency contributes to economic sustainability by lowering raw material costs, which is particularly beneficial for high-value metals such as titanium and cobalt-chromium alloys. Utilizing mechanical milling to generate feedstock powders for metal AM enables the creation of a sustainable powder production method by transforming chips of various materials into powders of different sizes (Fullenwider et al. 2019). The mechanical milling process operates without the need for elevated temperatures, resulting in considerably reduced energy consumption compared to conventional atomization methods (Fullenwider et al. 2019). Moreover, metal AM achieves a high material recovery rate of approximately 97% compared to traditional manufacturing methods, and it does not require significant tooling or molding operations (Ford and Despeisse 2016; Peng et al. 2018). Based on this, metal AM offers several advantages, and these advantages need to be thoroughly studied through sustainability assessments to understand their implications for resource efficiency and environmental impact.

Regardless of the anticipated future benefits and promising advantages of metal AM, there is still a lack of exploration from the perspective of sustainability assessment. Recent advancements have brought attention to the sustainability assessment for the deployment of metal AM techniques for various applications. Nevertheless, there is currently no comprehensive methodology that adequately addresses all the parts of the TBL, namely environmental, economic and social factors. Taddese et al. (2020) stated that several techniques that were implemented while developing the sustainability assessment lacked a direct indication of the weak stages in the life cycle of the AM system, most prominently in the social block. Most of the methods or techniques used to build up the assessment of sustainability are merely bounded to certain phases of the life cycle; thus, the findings about sustainability cannot be interpreted correctly for the overall integrated system (Meng et al. 2020; Zhang et al. 2022). Moreover, prior frameworks or tools for sustainability assessment fail to identify the specific areas of weakness throughout the entire life cycle of 3D-printed components in metal AM technology, highlighting the need for further refinements (Faludi et al. 2015; Huang et al. 2016; Tang et al. 2016). Hence, the opportunity to come up with an extensive assessment of sustainability related to metal AM is needed.

This work aims to address this research gap by providing a review of recent sustainability assessments of metal AM, focusing on papers that evaluate more than one sustainability dimension in the development of assessments. While most review papers on the sustainability assessment of metal AM have addressed broader areas, refining the focus in this manner will help evaluate the trends and identify the shortcomings of current sustainability assessments in metal AM. Without this refined focus on multiple sustainability dimensions, many gaps may be overlooked, hindering the development of improvements that consider all components of the TBL. With this context in mind, the primary objective of this article is to provide a thorough review and analysis of the recent sustainability assessment of metal AM. Investigating various themes that reveal the specific characteristics of sustainability assessments is essential for addressing the identified research gaps. Firstly, the review focuses on sustainability assessments as a whole, examining sustainability dimensions, state of indicators, integration of sustainability dimensions and circular economy (CE) domains. Subsequently, the review focuses on AM, covering the life cycle stages of AM, types of technologies or methods and their applications.

This paper presents a thorough review of the literature, uncovering significant insights related to its contributions. It begins by detailing the current and comprehensive state of sustainability assessment of metal AM and identifies key challenges and barriers to these assessments. This in-depth analysis spans a decade (2014–2024), highlighting developments in the field of sustainability assessments for metal AM over this period. The structure of the paper is as follows. Section 2 reviews key literature and theoretical foundations relevant to sustainability in metal AM. Section 3 explains the methodology used in this study, followed by Sect. 4 discusses the results of the findings. Section 5 outlines potential research trajectories that can help address the gaps identified in the current literature. It highlights key areas where further investigation is needed to advance the integration of sustainability assessments within the metal additive manufacturing field. Lastly, the study concludes with a summary of the main insights.

## Literature review

### Overview and core concept

This section offers background information on the topic and introduces key concepts. It begins with a definition and a detailed discussion of sustainability assessment, sustainability indicators and CE domain. The focus then shifts to metal AM, highlighting life cycle stages and types of technology.

The concept of sustainability assessment encompasses a methodology that guides decision-makers and policymakers in determining which actions to take or avoid for the betterment of societal sustainability (Sala et al. 2015). Over the past few decades, sustainability assessment has gained significant attention as a pivotal tool for achieving sustainable development. Numerous important and promising sustainability assessment initiatives have been undertaken across various engineering applications, including energy systems, mining and mineral industry, urban planning and water treatment (Sharifi 2021; Starkl et al. 2022; Agusdinata et al. 2023). Usman et al. (2024) emphasized that the manufacturing industry plays a crucial role in global greenhouse gas emissions, underscoring the urgent need for cleaner energy practices. As the world grapples with increasing challenges from climate change and environmental degradation, the manufacturing sector emerges as a critical arena for transformative action. The evolution of sustainability assessment is vital in these sectors for achieving sustainability and reducing carbon footprints. Consequently, manufacturing companies are increasingly seeking approaches, techniques, and frameworks to measure and evaluate their sustainability performance.

The advancement of metal AM over the years has been promising. Cooke et al. (2020) defined AM as the process of layer deposition through repetitive activity to generate the 3D model structure. The process of obtaining the 3D printed component starts from the digital archives, such as CAD documents or 3D scanning outcomes which produces the real object based on the input of materials. According to Peng et al. (2018), AM can be seen majorly utilized in rapid prototyping in aerospace engineering, automotive technology, manufacturing equipment production areas, health services and dental practice. The producer could have various models for each technology according to construction volume, processing speed, material specification, print resolution and layer increment (Huang et al. 2015). Kellens et al. (2017a, b) provide a comprehensive life cycle analysis showing that while the raw material extraction for metals can be more energy-intensive than for plastics, the longer service life and recyclability of metal parts offer a lower environmental impact over the product’s entire life cycle. Besides, a sustainability review by Frazier (2014) highlights how metal AM supports the production of high-performance components that offer long-term sustainability advantages in sectors where reliability and strength are crucial.

Anderson et al. (2021) highlight that employing metal AM technology for the production of metallic components presents numerous advantages compared to conventional manufacturing methods. According to Gu et al. (2012), metal AM offers the possibility of refurbishing parts that would typically be deemed irreparable, such as turbine blades, bearing seals, and shafts. Furthermore, Frazier (2014) notes that when adequately processed, the static mechanical properties of materials produced via metal AM can match those of traditionally fabricated metallic components. Additionally, Stock and Seliger (2016) emphasize that AM facilitates the continued use of aging products by enabling retrofitting and extending the operational lifespan of equipment. However, studies on the sustainability of this specific technology are limited, despite numerous pieces of evidence highlighting how metal AM can contribute to sustainability.

Next, the sustainability indicators are chosen to determine the sustainability performance in a quantifiable form. Huang and Wu (2020) provide an overview of sustainability indicators and their role in evaluating manufacturing processes, highlighting how these indicators serve as measurable criteria for assessing multiple sustainability dimensions, including environmental, economic and social aspects. Two types of indicators can be seen as follows:

- Qualitative indicators. These indicators require quantification through surveys or based on the experience of decision-makers.
- Quantitative indicators These indicators are measurable and calculable using standard measurement techniques and formulas.

The selection of indicators must adhere to several criteria. Currently, many techniques are available for selecting the indicators including the process of analysis, judgment, questionnaire, literature or guidelines (Tan et al. 2015; Merino-Saum et al. 2020; Reid and Rout 2020; Rossi et al. 2020). At present, challenges remain on how to determine the indicators and interpret these indicators at the process level. Several indicators have been invented, for instance, the Ford Product Sustainability Index (FORD PSI) (Schmidt and Taylor 2006). Global Reporting Initiative (GRI) Indicators (Tarquinio et al. 2018) and Dow Jones Sustainability Index (Schmutz et al. 2020). These sets can easily be accessed and altered based on the goal definition. However, the ready-made sets are not comprehended as the set is developed based on a wider scope and is not dynamic for an overall integrated system.

One of the key aspects covered in this analysis is the incorporation of the CE domain in developing sustainability assessments for metal AM. According to Morseletto (2020), CE should align closely with sustainable development, demonstrating how societal needs can be integrated within ecological limits by balancing environmental conservation, social equity and economic well-being. Metal AM components are often recyclable at the end of their life cycle, enabling a CE approach. In this context, a report by the Ellen MacArthur Foundation (2019) emphasizes that metal AM fits well into a CE framework because metals can be infinitely recycled without significant loss of quality, whereas plastics typically degrade with each recycling cycle. Thus, adopting CE principles, alongside technological advancements, has proven highly effective in reducing waste output and decreasing dependence on virgin natural resources, while also improving product efficiency (Khajuria et al. 2022). In this study, the five core domains of the circular economy include recycling, recovery, design, reduction and resource efficiency (Morseletto 2020).

For metal AM technology, the feedstock which is the metal components needs to be melted and deposited on a substrate at which layers by layers are constructed to produce the intended 3D shape (Cooke et al. 2020). The two major popular methods for this technology are direct energy deposition (DED) and powder bed fusion (PBF) (Ngo et al. 2018). Additionally, binder jetting (BJ) has garnered considerable interest in direct AM owing to its high productivity and ease of integration within the powder metallurgy industry (Li et al. 2020). Laser powder bed fusion (LPBF), direct metal laser sintering (DMLS) and selective laser melting (SLM) fall under the category of PBF processes (Mohd Yusuf et al. 2019). Additionally, wire and arc AM (WAAM), arc-fused spray deposition (AFSD) and shaped metal deposition (SMD) can be classified as technologies under DED. DED is the most convenient method in the current marketplace is a system called laser powder bed fusion (LPBF) due to the availability to build higher resolution with a standard layer thickness between 10 μm and 50 μm whereas the powder-fed DED printers manage to have about 250 μm layer thickness (Sames et al. 2016). In addition, fused deposition modeling (FDM) is an AM technique that involves the continuous extrusion of material through a nozzle while following a specified pattern to produce a part cross-section (Kellens et al. 2017a, b).

Pure metals are challenging to utilize in the AM industry due to their low mechanical properties (Basak and Das 2016). Consequently, various alloys are rapidly gaining popularity in the metal AM sector due to their significant potential from an industrial perspective. The most prevalent pre-alloyed materials currently under investigation are Fe-based, Al-based, Ti-based, Co-based and Ni-based alloys, with elements such as Cr and Mo exhibiting favorable alloying properties for printability (Basak and Das 2016). Table 1 presents detailed information on each of these alloys in the metal AM industry. SLM system is the most widely used method for producing these alloys, based on data from major AM companies in Germany and the United Kingdom (Cooke et al. 2020).

**Table 1:** Example of pre-alloyed in the metal AM industry

Type of pre-alloyed	Example
Fe-based	316L, 17-4PH, maraging steel 1.2709 (MS1/M300), H13 tool steel and Invar® 36
Ni-based	Specific Inconel® alloys such as IN 625, IN 718
Co-based	Co-Cr–Mo
Al-based	AlSi10Mg, AlSi9Cu3, AL 6061
Ti-based	Ti-6Al-4V

To fully comprehend the sustainability assessment of metal AM, it is crucial to examine all relevant inputs and outputs throughout each life cycle stage of an additively manufactured product (Kokare et al. 2023a). An initial step in this process involves gaining clarity on the distinct life cycle stages related to metal AM. Table 2 outlines the four life cycle stages considered in this study of metal AM (Ma et al. 2018).

**Table 2:** The life cycle stages of metal AM consist of four different stages

Stage	Description
Design	Centers on the design and planning of product architecture Three key tasks are carried out: creating the product’s CAD model, selecting the appropriate materials and designing the manufacturing process
AM	The fabrication process of the product Include upstream processes such as material extraction, electricity generation and pre-processing
Service	Encompasses the period from product delivery to the customer until the end of its life cycle or when it is no longer in use
End-of-life (EOL)	As products reach the end of their useful life, strategies are needed to maximize their remaining value. Common EOL approaches include recycling, repairing, remanufacturing, reuse, and disposal Some options of EOL approaches are recycle, repair, remanufacturing, reuse and disposal

## Methods

Before refining the selection strings, it is imperative to initially comprehend and establish the subject of interest. Using a broad topic in the search column might lead to an overwhelming number of documents and vague notions, which may hinder the thorough findings in the analysis. The search starts by finalizing two topics of interest in this study which are “sustainability assessment” and “metal additive manufacturing.” The search keywords for the article also included “3D printing,” “TBL,” and “life cycle assessment.”

The designations customarily practiced in tandem with the term AM have been developing over time, coherent with advancement of the technology. Several terms came in line with the evolution of AM such as: i) desktop manufacturing; ii) rapid prototyping; iii) rapid tooling; iv) 3D printing; and v) freeform fabrication. Due to the lack of standardized vocabulary in the field of AM, researchers face difficulties in identifying the most appropriate terminology in the published literature to ensure a comprehensive understanding for readers. The term “3D printing” commonly avails the nomenclature of marketers of the industry, communication media, decision-makers and the general public (Bourell 2016).

As mentioned earlier, this paper focuses on the sustainability assessment of metal AM. However, the search string does not specify metal AM exclusively, to avoid excluding relevant papers that may use different terminology. Using these keywords, relevant documents and articles were collected from the previously mentioned sources. The search focused on English-language publications from 2014 until 2024. Several databases, including Springer, ScienceDirect, Scopus, Sage and Emerald, were examined. The research strategy followed the framework designed by Okoli and Schabram (2010). Only papers related to metal AM were selected during the screening process.

Following the compilation of pertinent literature, qualitative content analysis (QCA) (Haapanen and Tapio 2016) was employed to identify various themes and categories associated with the sustainability assessment framework for metal AM. A total of 34 articles were initially retrieved. After screening titles and abstracts, followed by full-text evaluation, 18 studies were selected for final analysis. The extracted themes were categorized according to TBL dimensions: environmental, economic and social. Data were systematically extracted using Microsoft Excel to maintain consistency across the selected studies. Emerging patterns and research gaps were then synthesized to identify methodological trends and limitations in sustainability assessments related to metal AM. For data extraction purposes, information was gathered on the type of assessment method, system boundaries covered, scope of sustainability pillars, type of AM technology, number of indicators, integration among the covered scopes, circular economy domain and others. Drawing on the gathered data, a qualitative synthesis of methodologies and a combination of qualitative and quantitative analyses of the tools were conducted, and the findings were discussed. Additionally, the similarities and differences among the examined assessment tools were emphasized, and the areas for further development were identified.

### Current sustainability assessment of metal AM

Prior to reviewing and analysing the sustainability assessment of metal AM, a brief description of each method, as well as any integrated methods, is essential to enhance understanding and support practical application. The selected articles employ multiple assessment tools, which are either applied individually or integrated to provide a more comprehensive evaluation. This study exclusively centers on the sustainability evaluation of metal AM, encompassing two or more sustainability dimensions within its research framework. As a result, the application of the life cycle assessment (LCA) method can be analysed in conjunction with alternative methodologies, whether they are integrated or independent.

According to ISO 14040:2006, LCA can be referred to as a method of analysing the environmental burdens of any system starting with the initial phase until the end phase of the process of life stages. LCA is an established assessment method used to determine and offer comprehension of the environmental impact of particular products, systems and services over the entirety of their lifecycle (Hellweg and Milà i Canals 2014). For over three decades, research works and integrated approaches related to LCA have made this assessment the most chosen alternative for assessing the potential ecological effects of recent technologies (van der Giesen et al. 2020). The classic LCA is conducted through ISO 14040–14044 standards and the functional guidance can be found in several manuals (Baumann and Tillman 2004). This guidance gave the pre-existing directions on how to utilize the model and assess the retrospective of the environmental impacts after the product has been actively utilized commercially during the extended period.

Researchers employed several methods in developing sustainability assessments to ensure that a holistic and comprehensive sustainability impact can be obtained. One of the methods that is widely used with LCA is called life cycle costing (LCC). The development of LCC follows the same framework as the LCA study, with the purpose and scope being identical. It quantifies all the factors that significantly impact the costs throughout the life cycle, from the perspective of individuals, interested parties or involved stakeholders (Abdalla et al. 2021). However, the economic assessments were limited because crucial data, such as structural element constraints and inventory data from various sources, were unavailable. About the growth of LCC study in many field areas, there is still insufficient LCC literature that supports AM technology for decision-making processes. Considering that the economic impacts are strongly related to resource consumption and energy utilization in the deposition process, AM technology plays a major contribution in minimizing the expenses for case analysis with substantial scale, complicated configurations or custom-made models (Gouveia et al. 2022). AM contributes to economic sustainability by influencing cost factors across various life cycle stages. During the design phase, AM enables topology optimization and part consolidation, reducing the number of components required and minimizing assembly-related costs. This also reduces labor and inventory demands. In the production stage, AM eliminates the need for expensive tooling and allows for near-net-shape manufacturing, which significantly lowers raw material consumption and machining time. This is especially advantageous for high-value metals such as titanium or cobalt-chromium alloys.

Moreover, social life cycle assessment (S-LCA) is considered a highly effective method for evaluating social impacts from a life cycle perspective. This approach examines the social effects of products and services across their full life cycle, beginning with raw material extraction and continuing through to the EOL stage (Hannouf et al. 2024). Next, Environmental Impact Assessment (EIA) is a technique employed to assess the environmental impact of a particular project, enabling local authorities to gather insights on its ecological implications (Nadir and Ahmed 2023). This decision-making framework promotes sustainable development by proposing appropriate alternatives and mitigation strategies. There are several EIA conducted on AM processes that show that AM offers a better environmental impact compared to conventional manufacturing concerning material consumption and water usage (Agrawal and Vinodh 2020).

Safe and Sustainable-by-Design (SSbD) is a framework developed by the Joint Research Centre (JRC), which is currently being promoted through significant investments from the European Commission et al. (2022). This framework outlines a thorough methodology for assessing and establishing safety and sustainability standards for chemicals and materials throughout the innovation process (Moura et al. 2024). Next, multi-criteria decision analysis (MCDA) is applied in multi-criteria situations commonly encountered in the decision-making process of sustainability assessments (Ziemba 2022). Some methods classified under this category include Analytic Hierarchy Process (AHP), Technique for Order Preference by Similarity to Ideal Solution (TOPSIS) and entropy method.

The emergy framework has been utilized in developing sustainability assessments of mechanical manufacturing, specifically concerning processes and systems (Liu et al. 2018). This framework addresses the challenges of variability and objective quantification in environmental impact assessment (Cai et al. 2018). According to Odum (2007), the term “emergy” refers to the process of emergy analysis, which is also known as emergy accounting, evaluation, and synthesis. Gao et al. (2024) propose the emergy theory as a solution to the challenges encountered in the sustainability assessment modeling of metal AM systems.

The DMAIC (Define/Measure/Analyze/Improve/Control) methodology is another approach utilized during the development of sustainability assessments for metal AM. These systematic steps are employed to identify and address various types of issues, which are enhanced by the tools provided by Industry 4.0 (Rodriguez Delgadillo et al. 2022). Several other methods or assessments that can be identified from this analysis include quantitative assessment method, cost analysis, cost assessment, cost estimation, hazard assessment, human toxicity potential, machinability analysis, carbon emission assessment, the process-based cost model (PBCM) and the green index.

Table 3 illustrates the number of articles reviewed over the years, leading up to the results and analysis presented in the following section. A noticeable upward trend is evident from 2016, culminating in a peak of seven articles in 2023. As this study was conducted in mid-2024, an exact count of publications for that year is not available. Additionally, Table 3 shows the *Sustainability* and *Sustainable Materials and Technologies* published the highest number of articles (*n* = 3) related to the sustainability assessment of metal AM.

**Table 3:** Statistics of the reviewed articles, including publication year and source, within the research area

Publication Year	No of article
2024	4
2023	6
2022	3
2021	1
2020	-
2019	1
2018	1
2017	1
2016	1
2014–2015	-
Source in this research area	No of article
Sustainable Materials and Technologies	3
CIRP Journal of Manufacturing Science and Technology	1
Sustainable Production and Consumption	2
Journal of Manufacturing Processes	1
Journal of Cleaner Production	1
Procedia CIRP	1
Energy	1
Sustainability	3
Journal of Manufacturing Systems	1
The International Journal of Advanced Manufacturing Technology	2
International Design Engineering Technical Conferences and Computers and Information in Engineering Conference	2

## Result and discussions

This section presents the analysis of the sustainability assessment of metal AM. Table 4 provides a comparative overview of the sustainability assessment employed in metal AM studies. A total of 18 sustainability assessment methods related to metal AM were reviewed, as shown in Table 4. The majority of these assessments were based on LCA, with 13 out of the 18 assessments utilizing this approach. Closely following this, LCC was applied in 4 out of the 18 assessments. Notably, all papers that employed LCC also utilized LCA for their sustainability evaluations; however, these tools were applied independently (Manco et al. 2023; Catalano et al. 2023; Dias et al. 2022). This indicates a lack of comprehensive sustainability scores that integrate both environmental and economic factors. Furthermore, the Green Index is incorporated into both LCA and LCC assessments, serving as a performance indicator that provides comprehensive insights into the product’s sustainability (Manco et al. 2023). The implementation of LCA is frequently combined with other assessment methods, including PBCM (Dias et al. 2022), emergy analysis (Jiang et al. 2019) and cost analysis (Raoufi et al. 2022; Santiago-Herrera et al. 2023). Additionally, LCA has been integrated with various other assessment approaches, such as SSbD and hazard assessment (Moura et al. 2024), TOPSIS and entropy (Ahmed et al. 2024), as well as HTP and cost assessment (Ma et al. 2018).

**Table 4:** Comparison of sustainability assessments of metal AM, based on the method, sustainability dimensions, number of indicators, reference of indicators, integration of dimensions, sensitivity analysis and circular economy domain

No	Reference	Approach/ method	Sustainability dimensions			Number of indicators		Indicators reference		Integration of sustainability dimension		Sensitivity analysis		CE domains				
			Environmental	Economic	Social	< 10	> 10	Literature based	Expert approved	Yes	No	Yes	No	Recycling	Recovery	Reuse	Resource efficiency	Reduction
1	Moura et al. 2024	SSbD, LCA, hazard assessment	√		√	√		√			√		√				√	
2	Pusateri and Olsen 2024	LCA, LCC	√	√			√	√			√	√		√			√	
3	Gao et al. 2024	Emergy-based EIA	√	√		√		√		√			√				√	
4	Ahmed et al. 2024	TOPSIS, Entropy, LCA	√	√	√	√		√		√		√					√	
5	Gonçalves et al. 2023	LCA, LCC	√	√			√	√			√		√	√			√	
6	Manco et al. 2023	LCA, LCC, green index	√	√			√	√		√		√					√	
7	Salvi et al. 2023	Carbon emission assessment, cost analysis, machinability analysis	√	√		√		√			√		√				√	
8	Kokare et al. 2023b	LCA, LCC	√	√			√	√			√		√				√	
9	Catalano et al. 2023	Cost assessment, LCA	√	√		√		√			√		√				√	
10	Santiago-Herrera et al. 2023	LCA, cost analysis	√	√			√	√			√		√				√	
11	Raoufi et al. 2022	LCA, cost analysis	√	√		√		√			√	√					√	
12	Rodriguez Delgadillo et al. 2022	DMAIC Framework	√	√	√		√	√			√		√				√	
13	Dias et al. 2022	LCA, PBCM	√	√		√		√			√	√					√	
14	Wang et al. 2021	Emergy-based evaluation method	√	√		√		√			√		√				√	
15	Jiang et al. 2019	Emergy, LCA	√	√	√		√	√			√		√				√	
16	Ma et al. 2018	LCA, human toxicity potential, cost assessment	√	√	√	√		√			√		√				√	
17	Chan et al. 2017	Environmental impact analysis, economic analysis, social impact calculation	√	√	√	√		√			√		√				√	
18	Doran et al. 2016	Quantitative Assessment Method	√	√	√	√		√			√		√				√	

Integrating other sustainability evaluation methods such as PBCM, Emergy analysis, and hazard assessment is essential to achieve a more comprehensive understanding of sustainability impacts in metal AM. While LCA effectively captures environmental impacts based on material and energy flows, it does not provide detailed insights into economic costs, energy quality, or potential health and safety risks. The integration of PBCM allows for the simultaneous evaluation of both environmental and economic performance, which is particularly important in metal additive manufacturing due to high production costs and complex processing requirements. Emergy analysis contributes by accounting for the total energy investment, including both direct and indirect energy flows, offering a systemic view of resource efficiency. This is especially relevant in energy-intensive metal AM processes such as laser-based powder bed fusion. Hazard assessment complements the environmental and economic analyses by identifying occupational and environmental risks associated with exposure to fine metal powders, high temperatures, and potentially hazardous emissions. Although this multi-method approach provides a more holistic perspective, it introduces challenges such as increased data demands, methodological alignment, and complexity in interpreting results. Despite these difficulties, the integrated framework enhances the reliability and relevance of sustainability assessments for decision-making in research, industrial applications, and policy development.

From the perspective of sustainability dimensions, the inclusion of environmental and economic aspects was ranked highest, with 11 out of 18 assessments incorporating these elements. Following this, 6 out of 18 assessments included all three components of the TBL. The lowest ranking was assigned to assessments that focused solely on environmental and social dimensions, with only 1 out of 18 evaluations reflecting this combination. This trend indicates that, despite the broad acceptance of the TBL framework for sustainability, the incorporation of the social dimension in sustainability assessments of metal AM remains limited due to various factors.

The incorporation of various sustainability dimensions underscores the thoroughness of sustainability assessments in metal AM, with the number of indicators serving as an additional factor influencing their overall comprehensiveness. It is often recommended that a greater number of relevant indicators be utilized to provide a more complete view of sustainability performance. Among the sustainability assessments analysed, the majority were based on fewer than 10 indicators (11 out of 18), while the remaining assessments employed more than 10 indicators (7 out of 18). The inclusion of a broader range of indicators is essential for effectively evaluating trade-offs among environmental, social and economic dimensions. Munda (2016) emphasizes the significance of multi-dimensionality in sustainability assessments, indicating that reliance on too few indicators may limit the assessment’s ability to capture the full spectrum of impacts. Furthermore, rather than depending on generic and broad sustainability indicators, it is crucial to develop, prioritize and implement specific indicators tailored to individual industries (Hsu et al. 2017). Indicators derived from expert validation generally possess greater credibility among stakeholders. Validation of indicators by field experts enhances their reliability and acceptance within the industry, fostering trust in the sustainability assessment process. This aspect is particularly critical when decisions are made that could substantially influence environmental, economic, and social outcomes. Additionally, experts play a vital role in identifying and prioritizing key performance indicators essential for assessing sustainability within a specific context. This focused approach streamlines the assessment process and directs efforts toward the most impactful areas, thereby enhancing overall effectiveness. Notably, all sustainability assessments of metal AM rely solely on sustainability indicators derived from literature reviews without undergoing validation.

Next, the integration of sustainability dimensions in the reviewed literature is examined. The analysis revealed that, among the sustainability assessments for AM, only a limited number of studies incorporated these dimensions into a unified sustainability score (3 out of 18). Notably, the study conducted by Ahmed et al. (2024) is the sole paper that addresses all components of the TBL, successfully integrating them into a comprehensive score. Integrating environmental, economic and social dimensions into a single sustainability score is necessary for a holistic evaluation of sustainability performance, allowing more informed decision-making and better trade-off analysis. This approach helps balance conflicting priorities among the three dimensions, offering a comprehensive view of sustainability (Moldan et al. 2012). A unified score simplifies complex data, making it easier for stakeholders to assess sustainability in a standardized manner (Singh et al. 2012).

From the perspective of CE domains, all reviewed papers fall under the category of resource efficiency. Additionally, some of these papers also incorporate the domains of resource efficiency and recycling in their sustainability assessments of metal AM (Gonçalves et al. 2023; Pusateri and Olsen 2024; Ahmed et al. 2024). The subject of recycling should be included as one of the topics in the sustainability assessment of metal AM. The rationale for this inclusion is that recycling promotes resource conservation by recovering valuable metals from EOL products or production waste. Recycling scrap generated from SLM in powder bed fusion PBF processes can result in as much as a 50% decrease in the Abiotic Resource Depletion impact score (Walachowicz et al. 2017). Additionally, unfused powder in SLM can potentially be reprocessed, allowing for a recovery rate of up to 30% (Wurst et al. 2023). This approach decreases the demand for new raw materials, helping to preserve natural resources and reduce environmental harm. It aligns with the CE framework, which promotes sustainable resource utilization (Colorado et al. 2020).

Table 5 displays the sustainability assessment of metal AM, including information on the life cycle stages covered by the sustainability dimensions, the type of technology or method used in AM, type of metal employed and the applications where sustainability assessment is applied. Within the reviewed sustainability assessments of metal AM, only a limited number encompass the design, AM, use and EOL stages (2 out of 18). All reviewed papers include the AM stage as their scope boundary, focusing on the sustainability impacts associated with the manufacturing process of metal AM. The design stage is also included in the sustainability assessments of metal AM (5 out of 18). Additionally, both the service and EOL stages are covered in the evaluation of sustainability impacts related to metal AM (3 out of 18). Incorporating all life cycle stages facilitates the identification of potential sustainability improvements throughout the entire process, rather than concentrating solely on isolated phases.

**Table 5:** Comparison of sustainability assessment of metal AM (additional parameter)

No	Reference	Approach/method	Life cycle stages covered by sustainability assessment				AM technology/method	Type of metal used	Application
			Design	AM	Service	EOL			
1	Moura et al. 2024	SSbD, LCA, hazard assessment		√			DED	Fe_81_Cr_16_Al_3_, Al-TiC, Al-TiB_2_, MoCu, CuCr	Materials for metal AM
2	Pusateri and Olsen 2024	LCA, LCC	√	√	√	√	WAAM	-	Repairing component
3	Gao et al. 2024	Emergy-based EIA		√			SLM,	316L powder	New part
4	Ahmed et al. 2024	TOPSIS, Entropy, LCA		√			DMLS, AFSD	Ti-6Al-4 V	New part
5	Gonçalves et al. 2023	LCA, LCC	√	√		√	SLM	Ti-6Al-4 V, M300 steel powder	New part
6	Manco et al. 2023	LCA, LCC, green index	√	√	√		Not specified	Ti-6Al-4 V	New part
7	Salvi et al. 2023	Carbon emission assessment, cost analysis, machinability analysis		√			WAAM	IN-625	-
8	Kokare et al. 2023b	LCA, LCC		√			WAAM, SLM	Metal powder	New part
9	Catalano et al. 2023	Cost assessment, LCA		√			SLM	Ti-6Al-4 V	-
10	Santiago-Herrera et al. 2023	LCA, cost analysis		√			DED	Ti-6Al-4 V	Materials for metal AM
11	Raoufi et al. 2022	LCA, cost analysis		√			LPBF, BJ	316L Stainless Steel	New part
12	Rodriguez Delgadillo et al. 2022	DMAIC Framework	√	√			BJ	316L, AlSi1_0_Mg,	New part
13	Dias et al. 2022	LCA, PBCM		√			SLM	316L powder	New part
14	Wang et al. 2021	Emergy-based evaluation method		√			WAAM	316L powder	New part
15	Jiang et al. 2019	Emergy, LCA		√			DED	AISI 4140	New part
16	Ma et al. 2018	LCA, human toxicity potential, cost assessment	√	√	√	√	FDM	Unspecified	New part
17	Chan et al. 2017	Environmental impact analysis, economic analsyis, social impact calculation		√			DED, SLM	Ti-6Al-4 V	New part
18	Doran et al. 2016	Quantitative Assessment Method		√			DED	Unspecified	New part

Based on the AM technologies and methods applied in the reviewed papers, SLM emerges as the most frequently utilized machine for sustainability studies (6 out of 18). This is followed by DED (5 out of 18), WAAM (4 out of 18) and BJ (2 out of 18). The remaining AM technologies and methods were addressed as research subjects only once. SLM and WAAM have become central to the sustainability assessment of metal AM for a variety of reasons. SLM is widely recognized for its capability to create intricate geometries while minimizing material waste, which positions it as a valuable candidate for sustainability evaluations. By employing a powder bed fusion approach, SLM achieves high rates of material utilization, which is vital for conserving resources (Gao et al. 2015). In a similar vein, WAAM demonstrates material efficiency by utilizing wire feedstock to fabricate larger components with reduced waste when compared to conventional manufacturing techniques (Rodrigues et al. 2019). Gaining insights into the material efficiency of these technologies is crucial for assessing their overall sustainability. Although metal additive manufacturing continues to advance rapidly, the current body of sustainability research is predominantly centered on laser-based techniques such as SLM and DED, while other methods like BJ remain comparatively underexplored. This limited focus results in an incomplete understanding of sustainability performance across the full range of metal AM technologies. BJ, for example, presents distinct advantages, including faster build rates, greater scalability, and reduced thermal energy input, which may lead to different environmental and economic outcomes compared to fusion-based methods. However, the scarcity of empirical studies on critical aspects such as energy use, powder recyclability, emissions and post-processing requirements in BJ hinders a comprehensive evaluation of its sustainability potential. To address this gap, future research should prioritize comparative assessments that examine BJ in relation to other AM processes, utilizing standardized methodologies such as LCA, LCC and SLCA. Such studies should incorporate process-specific variables including binder composition and toxicity, sintering energy demands, material utilization rates and overall production efficiency. Broadening the scope of technology assessment would contribute to more balanced and inclusive sustainability evaluations, enabling industries to make informed strategic decisions on equipment acquisition, material choices, production planning and environmental compliance.

Based on the analysis of sustainability assessment applications, the majority of assessments focus on evaluating new metal parts produced by metal AM (13 out of 18 studies). Additionally, two studies specifically examine the sustainability of materials used in metal AM, concentrating on the processes involved in producing these metals prior to their application in component manufacturing (Kokare et al. 2023a, b; Moura et al. 2024). Furthermore, one study investigates the sustainability impacts of repairing existing components rather than producing new parts (Chan et al. 2017). Overall, the diverse applications and benefits of AM position it as a transformative technology across various industries, driving advancements in efficiency and sustainability.

The findings of this review offer several valuable and practical implications. The sustainability assessment of metal AM was critically analysed. Firstly, review papers can help identify best practices in sustainability assessments by showcasing successful case studies and examples of effective implementation within the metal AM sector. This review can outline the advantages and limitations of the sustainability assessments from the studies reviewed, allowing stakeholders to better understand which methodologies are most effective and reliable for specific applications (refer to Table 6). These papers can compare the various sustainability assessments applied to metal AM, highlighting their strengths and weaknesses. Such comparisons can guide researchers and industry practitioners in selecting the most appropriate methods for their specific contexts. By disseminating knowledge on sustainability assessments, review papers can also explore how metal AM technologies align with CE principles, including resource efficiency and recycling. This alignment is crucial for promoting sustainable manufacturing practices and reducing the overall environmental footprint.

**Table 6:** Strengths and disadvantages of sustainability assessment in metal AM

No	Reference	Details	Strength	Disadvantage
1	Moura et al. 2024	This study evaluates the safety and environmental impact of nano-enabled multifunctional materials for developing safer and more sustainable alternatives in metal AM	It successfully balances the dual goals of environmental sustainability and health safety, showcasing a holistic approach	The paper is designed solely for the production of materials for metal AM, which excludes all other significant processes in the AM stage
2	Pusateri and Olsen 2024	This study provides a cradle-to-grave assessment focusing on the environmental and economic aspects of metallic components manufactured using AM	This study illustrates how LCA and LCC provide vital ex-ante insights that are essential for strategic implementation	There is a disparity between the reliance on standardized data from the Ecoinvent 3.9.1 database for conventional practices and the dependence on primary data from industrial partners for the WAAM alternative
3	Gao et al. 2024	This paper establishes a unified dimensional evaluation model for AM, integrating processing quality, cost, energy and material considerations using an emergy-based approach	This study thoroughly examines the attributes of various AM processes, develops a cohesive evaluation framework for the sustainable assessment of AM system and presents solutions to address critical factors influencing the sustainability of AM	No social evaluations were discussed
4	Ahmed et al. 2024	This paper evaluates environmental, economic and social dimension of AFSD and DMLS	This paper presents an initial framework for integrating the TBL components in metal AM	The assessment is limited to the AM stage
5	Gonçalves et al. 2023	This paper delivers an in-depth evaluation of the environmental and economic sustainability impacts of metal AM within two key industries: industrial machinery and aerospace	This paper integrates EOL stages into the sustainability assessment, enhancing the understanding of recycling’s role in achieving a closed-loop system	Lack of comprehensive indicators that specifically assess the sustainability performance of metal AM across various industries
6	Manco et al. 2023	The proposed model utilizes a parametric analysis that takes into account six strategic variables and integrates both LCA and LCC	Green Index effectively integrates sustainability scores for environmental and economic factors, strengthening the framework for evaluating the components of the TBL in a comprehensive manner	The model simplifies the supply chain structure by omitting a detailed network of raw material suppliers, distribution hubs, and centers, while also disregarding the influence of uncertainty
7	Salvi et al. 2023	This paper assesses the environmental impact, economic factors, and machinability of Inconel 625 (IN 625)	The incorporation of machinability factors into the sustainability assessment could facilitate the development of a more comprehensive methodology	There is a lack of integration of methodologies to comprehensively assess the overall sustainability score of metal IN 625
8	Kokare et al. 2023b	This paper evaluates the environmental impact and production costs linked to the fabrication of a marine propeller utilizing WAAM and SLM methods	This study examines the influence of post-processing operations, such as machining processes, on achieving final in WAAM	The assessment is limited to the AM stage
9	Catalano et al. 2023	No social evaluations were discussed		
10	Santiago-Herrera et al. 2023	This paper evaluates how the deposition rate in Wire Arc Additive Manufacturing (WAAM) affects environmental and economic sustainability indicators	This paper underscores the importance of incorporating the deposition rate as a key factor in the development of sustainability assessments	The assessment is limited to the AM stage
11	Raoufi et al. 2022	This paper evaluates the economic and environmental performance of metal AM in the production of a microscale chemical reactor made from 316L stainless steel	Sensitivity analysis employs can verify models by examining how anticipated changes in inputs correspond with actual variations in outputs	The assessment is limited to the AM stage
12	Rodriguez Delgadillo et al. 2022	This paper proposes a framework based on the DMAIC methodology that includes key performance indicators (KPIs)	It provides a comprehensive assessment of the TBL concept	Exclusion of service and EOL stages
13	Dias et al. 2022	This paper proposes an emergy-based approach to assess the environmental impacts and economic costs associated with the SLM manufacturing process	Emergy links economic value to environmental impact, facilitating a more integrated sustainability assessment that encompasses both ecological and economic dimensions	The assessment is limited to the AM stage
14	Wang et al. 2021	This paper assesses the economic and environmental potential of WAAM using a detailed PBCM and LCA through a cradle-to-grave approach	Sensitivity analysis helps break down complex models by focusing on key inputs, making the model easier to interpret and apply	The assessment is limited to the AM stage
15	Jiang et al. 2019	This study developed an emergy-driven LCA approach to evaluate the sustainability of Laser Engineered Net Shaping (LENS)	It offers insights into the sustainability of systems over time by evaluating how effectively energy and resources are utilized in production processes	Due to challenges in data collection, the social impact assessment in the case study only addresses human health damages
16	Ma et al. 2018	This paper develops comprehensive sustainability assessment of AMP, incorporating key stages such as design, manufacturing, usage and EOL management	By analysing all life cycle stages, it becomes easier to identify areas for improvement in sustainability	Given the inherent complexity of social sustainability, relying on a single aspect indicator is insufficient for a comprehensive evaluation of overall social sustainability performance
17	Chan et al. 2017	This study evaluates TBL components essential for assessing the sustainability of metal-based AM	It provides a comprehensive assessment of the TBL concept	Absence of integration among all sustainability dimensions
18	Doran et al. 2016	This study presents a method for assessing various sustainability metrics for components of varying sizes produced using AM	It provides a comprehensive assessment of the TBL concept	Absence of integration among all sustainability dimensions

## Potential research trajectory

From the analysis of the results and discussion, several gaps and challenges in sustainability assessment for metal AM have been identified and explored. This section outlines potential research directions aimed at addressing these identified gaps in the literature. As the sustainability assessment of AM continues to progress, there is a growing need to incorporate environmental considerations into the economic and social aspects of sustainability. This will enable a comprehensive understanding of sustainability assessment. Without empirical research data to substantiate these claims, it becomes challenging to establish the credibility of AM in the sustainability that covers TBL perspectives which are environmental, economic and social. Currently, there are recognized methods; notably LCA and LCC for the development for environmental and economic pillars, respectively.

However, the social aspect is often overlooked while developing a comprehensive sustainability assessment of metal AM despite the absence of firmly established life cycle techniques up to this point (Ribeiro et al. 2020). Despite the introduction of SLCA to analyse the social effects of products and systems, there is a significant gap in understanding the connection between metal AM and SLCA as the literature involved in the early phase focused on discussions and reviews rather than the true assessment involving the full life cycle stages (Huang et al. 2013). Referring to the earlier social studies, the focal point was given to a part of the cycle phase that involved design and production due to complicated attributes originating from social aspects. Moreover, the growing need for specialized training and upskilling programs to close the competence gap is frequently overlooked in current sustainability assessments. Broader community-level impacts in the regional employment dynamics or local resource consumption are also rarely considered despite their relevance to social sustainability. To overcome these gaps, future studies should consider participatory methodologies like expert consultations, stakeholder-based surveys, or the Delphi method to derive indicators that are socially meaningful and grounded in practical realities (Parent et al. 2010). By integrating these approaches, assessments of social sustainability in the metal additive manufacturing sector can better capture the concerns, expectations and risks experienced by diverse stakeholder groups throughout the value chain.

About the growth of LCC study in many field areas, there is still insufficient LCC literature that supports metal AM technology for decision-making processes. Considering that the economic impacts are strongly related to resource consumption and energy utilization in the deposition process, AM technology plays a major contribution in minimising the expenses for case analysis with substantial scale, complicated configurations or custom-made models (Gouveia et al. 2022). However, the economic assessments were limited by the absence of crucial data, including standardized practices for the evaluated alternatives, a lack of AM-specific processing details and inconsistencies in the sources of inventory data. Given this gap, sustainability assessments of metal AM should incorporate Artificial Intelligence (AI) systems and techniques. Methods such as fuzzy logic, artificial neural networks (ANN) and genetic algorithms (GA) can enable the integration of all TBL components, resulting in a more holistic sustainability evaluation of metal AM. Sabaghi et al. (2016) applied fuzzy-inference techniques (SAFT) to address the presence of ambiguities and unknowns during the process of quantifying the sustainability of the process. The findings indicated the developed technique is a more convenient and simplified approach given that it is autonomous in using fuzzy rules from the fuzzy rule-base method. A model called FELICITA (Fuzzy Evaluation for Life Cycle Integrated Sustainability Assessment) was developed by Kouloumpis and Azapagic (2018) to evaluate the sustainability of multiple systems by integrating the findings of LCA, LCC and SLCA. This model can resolve the challenges presented by the vagueness of data related to sustainability indicators or inputs without influencing the overarching outcomes. The key advantage of this model is it can be employed to discern relative variances across options contemplated which helps during the identification of the improvement. These encompass the necessity for carefully defining the rules and membership functions as the general fuzzy inference system is sensitive and dependent on one another. Over the past several decades, the combination of fuzzy systems and hybrid derivations can emulate the conventional human capacity for reasoning with computational efficacy (de Campos Souza 2020). There are two mainly focused hybrid systems based on the fuzzy system and artificial neural networks which are the neuro-fuzzy system (NFS) and fuzzy neural network (FNN). These systems have been assimilating theories and concepts through the advancement between data processing and neural networks in developing exceptionally precise systems to construct paradigms according to rules, expert systems, classifiers and universal approximators. The development of the fuzzy logic approach over the years has seen this method combined with several machine learning techniques. de Campos Souza (2020) concluded that NFS are more reliable in developing a system that has a high degree of precision and adequate level of understandability for various sectors in economic studies and scientific disciplines. Besides, NFS is capable of assessing the sustainability performance level of the additively manufactured products between “sustainable” and “not-very-sustainable.” Hence, the weak spots throughout the entire life cycle can be detected and adjusted for the presented life cycle.

Integrating life cycle stages into the sustainability assessment of metal AM is essential for determining the overall sustainability of this technology. The discussion reveals a significant gap in studies that encompass the design, AM, use and EOL stages. Notably, approximately 80% of sustainability impacts are established during the design phase, highlighting the need for researchers to focus on the design process within the manufacturing industry and to advocate for new strategies that align with sustainability objectives [14]. Many recent studies on the sustainability assessment of metal AM have overlooked the design stage, which has a profound influence on sustainability over time. For instance, the economic sustainability assessment should consider expenses associated with the design phase, including labour and utility costs. Moreover, metal AM plays a crucial role in the EOL stage, particularly concerning recycling. Metal AM processes often generate excess material during production, and effective recycling methods can recover these unused powders, minimizing waste and enabling their reuse in new production cycles. Integrating recycling into the EOL of metal AM components supports the development of closed-loop systems, where materials are continuously reused. This approach aligns with CE principles, promoting sustainability and reducing waste. By addressing these aspects, metal AM can significantly contribute to sustainability goals and responsible resource management at the EOL stage.

One of the central promises of the CE in metal AM is the reduction of material waste; however, this benefit can be compromised by the high energy requirements of metal printing processes, especially when non-renewable energy sources are used. In such cases, the environmental benefits gained from material efficiency may be offset by the carbon footprint of energy consumption. Another overlooked area in current assessments is EOL stage of metal AM components. The recyclability of complex, multi-material alloy parts remain uncertain, as challenges persist in separating and processing these materials efficiently. These limitations point to a broader need for consistent metrics and comprehensive life cycle inventory data that can accurately evaluate aspects such as powder reuse rates, recycling feasibility, and EOL pathways. Moving forward, research should work to clarify these unresolved issues and establish clear criteria to determine when and under what conditions metal AM aligns with circular economy goals in a truly sustainable manner.

The principles of the CE are gaining prominence in sustainable manufacturing. However, their integration into the sustainability assessment of metal AM remains relatively underdeveloped. CE strategies such as improving material efficiency, enabling design for reuse, implementing closed-loop recycling and extending product lifespans are well aligned with the technical potential of AM. Despite this compatibility, these principles are not yet consistently reflected in current assessment frameworks. Recent studies have started to examine how metal AM can contribute to CE goals. For instance, Javaid et al. (2021) highlighted how AM facilitates material circularity through practices such as on-demand production, remanufacturing-oriented design, and the reuse of metal powders. These capabilities help lower dependence on virgin raw materials and reduce production waste, particularly in powder bed fusion processes where unused powder can be recovered and reused if it meets certain quality standards.

From an environmental perspective, applying CE practices in AM can reduce the extraction of natural resources, lower manufacturing scrap, and minimize emissions from transportation by supporting localized and resource-efficient production (Gebler et al. 2014). In addition, AM eliminates the need for traditional tooling and supports distributed manufacturing approaches, which can result in shorter supply chains and reduced operational costs. Despite these promising synergies, several limitations continue to hinder the full adoption of CE principles in sustainability assessments of metal AM. Most LCA primarily focus on environmental outcomes, while social and economic co-benefits are often overlooked. Furthermore, EOL strategies for AM-produced parts, especially those made from complex or multi-material metal alloys, are insufficiently addressed. This creates uncertainties regarding their recyclability and long-term circularity. In summary, CE principles have the potential to enhance the balance of environmental, economic and social aspects within sustainability assessments of metal AM. To fully realize this potential, future research should develop comprehensive models that quantify CE contributions across all three pillars of the triple bottom line. These models should also explore the trade-offs and practical challenges associated with powder reuse, energy intensity, and EOL considerations.

Future research should focus on incorporating a greater number of indicators with assigned weights for the sustainability assessment of metal AM. Different sustainability dimensions often encompass various indicators. Weighting these indicators ensures a balanced evaluation that reflects the relative importance of each dimension, leading to a more comprehensive understanding of overall sustainability. However, the majority of studies rely on reference-based indicators without the intention of developing expert-approved metrics. Such reference-based indicators may not accurately capture the unique challenges and priorities of the metal manufacturing industry. In contrast, expert-approved indicators are typically formulated through a thorough understanding of local conditions and stakeholder needs, rendering them more relevant and applicable.

## Conclusion

The ongoing rise in the number of sustainability assessments published in the field of metal AM technology is characterized by a lack of comprehensive information and insufficient documentation of the development of sustainability assessments encompassing environmental, economic and social aspects. This creates an urgent necessity to evaluate and analyse emerging sustainability assessment of metal AM through the lens of the TBL framework. This study aims to bridge the identified gaps by offering a comprehensive review and analysis of the methodological advancements and developments in sustainability assessment of metal AM published between 2014 and 2024.

The analysis conducted contributes to the existing body of knowledge by providing the latest insights, identifying research gaps, and highlighting progress and future directions in this area. It clearly demonstrates that the majority of sustainability assessments of metal AM rely primarily on LCA and LCC. However, there has been little effort to integrate environmental, economic, and social, for a more comprehensive assessment. Additionally, most assessments focus only on the AM stage, neglecting the design, use, and EOL stages, despite each stage significantly influencing sustainability outcomes. Furthermore, many sustainability indicators are based on references without applying weights, which limits their effectiveness in achieving long-term sustainability goals. By emphasizing specific indicators, organizations can better align their assessments with broader sustainability objectives.

Additionally, this paper is expected to assist the AM community in local industries by evaluating the sustainability of metal 3D-printed components using appropriate assessments and methods for specific life cycle stages of metal AM. It also aims to encourage organizations, policymakers and the AM community to adopt sustainability guidelines and frameworks by showcasing the technology as a sustainable solution. This will lead to improved resource efficiency, more effective production systems, and the adoption of innovative business models.

## Data Availability

The dataset used to analyze this study are available from the corresponding author upon reasonable request.
